# Accurate identification of Enterococcus lactis causing bacteraemia by matrix-assisted laser desorption ionization-time of flight mass spectrometry

**DOI:** 10.1099/jmm.0.001995

**Published:** 2025-04-04

**Authors:** Marhami Fahriani, Geoffrey W. Coombs, Princy Shoby, Haley Hood, Denise A. Daley, Christopher A. Mullally, Shakeel Mowlaboccus

**Affiliations:** 1 School of Medical, Molecular and Forensic Sciences, Murdoch University, Murdoch, Western Australia, Australia; 2 Department of Microbiology, PathWest Laboratory Medicine-WA, Fiona Stanley Hospital, Murdoch, Western Australia, Australia; 3 Australian Group on Antimicrobial Resistance, Fiona Stanley Hospital, Murdoch, Western Australia, Australia; 4 School of Biomedical Sciences, The University of Western Australia, Perth, Western Australia, Australia

**Keywords:** bacteraemia, *Enterococcus faecium*, *Enterococcus lactis*, MALDI-TOF MS

## Abstract

**Introduction.***Enterococcus faecium* clade B has recently been re-classified as *Enterococcus lactis*. Although *E. lactis* was previously associated with food products and probiotics, the recent re-classification has prompted the need for the accurate identification of this species and re-interpretation of its disease-causing ability. Since the re-classified *E. lactis* can currently only be identified by molecular techniques such as whole-genome sequencing, we constructed a MALDI Biotyper^®^ custom database to rapidly identify and differentiate *E. lactis* causing bacteraemia from *E. faecium*.

**Hypothesis/Gap statement.** The re-classification of *E. faecium* clade B as *E. lactis* warrants the development of rapid and accurate identification methods to distinguish these species, particularly in clinical settings where *E. lactis* may be misidentified as *E. faecium*.

**Aim.** The aim of this study was to construct a MALDI Biotyper^®^ custom database to rapidly identify and differentiate *E. lactis* causing bacteraemia from *E. faecium*.

**Methodology.** A total of 97 enterococcal isolates, including 38 *E. lactis*, 51 *E. faecium* and 8 non-*E. faecium* non-*E. lactis* enterococci (*E. avium*, *E. casseliflavus*, *E. cecorum*, *E. durans*, *E. faecalis*, *E. faecium*, *E. gallinarum*, *E. lactis*, *E. mundtii* and *E. raffinosus*) were investigated. Whole-genome sequence analysis was used to confirm the species of each isolate. A MALDI Biotyper^®^ in-house database was constructed using 29 *E. lactis* isolates and the ethanol/formic acid/acetonitrile preparation protocol. The in-house database was validated using the 97 enterococcal isolates and the extended direct transfer preparation protocol.

**Results.** Our in-house database correctly identified all isolates at the species level, including the *E. lactis* isolates, all of which were misidentified as *E. faecium* by the BioTyper^®^ MBT Compass reference library (2022). Of the 38 *E. lactis* isolates, 84.2% (*n*=32) were identified at the high probable species level (score ≥2.300), while the remaining 15.8% (*n*=6) were identified at the probable species level (score 2.000–2.299). Similarly, all *E. faecium* isolates (*n*=51) were accurately identified, including 84.3% (*n*=43/51) identified at the high probable species level and 15.7% (*n*=8/51) identified at the probable species level.

**Conclusion.** Our study provides a ready-to-use custom MALDI spectral database that can be implemented in clinical diagnostic and research laboratories to accurately identify *E. lactis,* which is currently misidentified as *E. faecium* by the standard spectrum database available on commercial platforms.

## Background

*Enterococcus faecium*, a Gram-positive bacterium, is a member of the ESKAPE organisms. ESKAPE consists of six virulent nosocomial bacterial pathogens (*E. faecium*, *Staphylococcus aureus*, *Klebsiella pneumoniae*, *Acinetobacter baumannii*, *Pseudomonas aeruginosa* and *Enterobacter* species) that have developed resistance mechanisms to multiple classes of antibiotics [1]. In 2024, the World Health Organization included vancomycin-resistant *E. faecium* on the high-priority list of bacteria, for which it is crucial to conduct research and develop strategies to prevent and control antimicrobial resistance [2]. Vancomycin-resistant *E. faecium* poses a public health challenge due to persistent infections and multidrug resistance, which limit treatment options for *E. faecium* infections [3].

Based on whole-genome sequences, *E. faecium* isolates are categorized into two phylogenetically distinct clades: clade A isolates, which are typically hospital-associated, and clade B isolates, which are typically community-associated [4]. Clade A isolates are further divided into subclade A1, consisting of clinical isolates, and subclade A2, consisting of animal-associated isolates. Unlike the multidrug-resistant clade A isolates, the non-hospital clade B isolates are typically susceptible to antibiotics. Furthermore, clade B isolates, often isolated as commensals, have fewer virulence genes and mobile genetic elements than subclade A1 isolates and thus have a smaller genome size [5].

Recently, *E. faecium* clade B isolates have been reclassified as a different species, *Enterococcus lactis* [4]. *E. lactis*, first reported in 2012 from Italian raw milk cheese, is closely related to *E. faecium* with 99.4% similarity based on 16S rRNA gene sequence analysis [6]. *E. lactis* is frequently isolated in milk and dairy products [6,7]. The benefits and safety of *E. lactis* as a probiotic product for humans have previously been studied. The probiotic *E. lactis* strain IITRHR1 has a cytoprotective potential against drug-induced liver injury [8], and the probiotic *E. lactis* strain IW5 has anticancer properties against several human cancer cell lines [9]. *E. lactis* may also inhibit the growth of pathogenic bacteria, including *Escherichia coli* O26, *S. aureus*, *Bacillus cereus*, *K. pneumoniae*, *Shigella flexneri*, *Streptococcus mutans* and *Listeria monocytogenes* [9]. In the food industry, *E. lactis* has been used as a starter [10], probiotic culture [11] and animal feed additive [12]. Although previously presumed safe, *E. lactis*, which is susceptible to ampicillin, can still cause severe disease in humans [13]. Therefore, due to potential safety concerns, in 2022, *E. lactis* was removed from the European Food Safety Authority’s Qualified Presumption of Safety [14]. Furthermore, *E. lactis* can harbour antimicrobial resistance genes – for example, *E. lactis* strain E843, isolated in China from faecal samples of pigs, harbours the *poxtA* gene, which encodes resistance to the last-line drug linezolid [15].

In Australia, a nationwide antimicrobial resistance surveillance programme on enterococcal bloodstream infections, known as the Australian Enterococcal Surveillance Outcome Programme (AESOP), has been performed by the Australian Group on Antimicrobial Resistance (AGAR) since 2013. The programme involves 33 laboratories servicing 55 hospitals from all Australian states and mainland territories. In addition to antimicrobial susceptibility testing, whole-genome sequencing (WGS) is performed on all isolates identified by the laboratories as *E. faecium*. In AESOP 2021, of the 1,297 enterococcal bloodstream infections reported, 523 (40.3%) were identified as *E. faecium* bacteraemia cases by the AGAR laboratories. However, WGS identified 28 (2.16%) *E. lactis* isolates that were incorrectly reported as *E. faecium* by the laboratories [16]. Similarly, in AESOP 2022, 29 of the 1,535 (1.89%) enterococcal isolates were misidentified as *E. faecium* by the AGAR laboratories and were confirmed to be *E. lactis* following WGS [14].

In Australian diagnostic microbiology laboratories, matrix-assisted laser desorption ionization-time of flight mass spectrometry (MALDI-TOF MS) is frequently used for bacterial species identification [17]. MALDI-TOF MS is a cost-effective and easy-to-perform assay that accurately identifies pathogenic organisms with minimal hands-on time and optimal turnaround time [18]. However, the two MALDI-TOF MS systems used in diagnostic microbiology laboratories, the MALDI Biotyper^®^ (Bruker Daltonics, Germany) and VITEK^®^ MS (bioMérieux, France), do not have *E. lactis* reference spectra in their databases and consequently are not able to distinguish *E. lactis* from *E. faecium* [17]. In this study, we aim to construct a MALDI Biotyper^®^ in-house database to rapidly identify and differentiate *E. lactis* from *E. faecium*.

## Methods

### Bacterial isolates

A total of 97 isolates representing ten different *Enterococcus* species (*E. avium*, *E. casseliflavus*, *E. cecorum*, *E. durans*, *E. faecalis*, *E. faecium*, *E. gallinarum*, *E. lactis*, *E. mundtii* and *E. raffinosus*) were included. All isolates were cultured on blood agar and incubated at 37 °C overnight. *E. cecorum* was incubated in the presence of 5% CO_2_. The clinical * E. faecium* and *E. lactis* isolates were obtained from bacteraemia cases reported in the 2020 and 2021 AESOP surveys. The *E. faecalis* (ATCC^®^ 51299) and *E. casseliflavus* (ATCC^®^ 700327) reference strains were obtained from the American Type Culture Collection (ATCC). The *E. lactis* reference strain KCTC 21015 was obtained from the Korean Collection for Type Cultures (KCTC).

### Whole-genome sequencing

Bacterial genomic DNA was extracted using the DNeasy^®^ Blood and Tissue kit (Qiagen, Germany) according to the manufacturer’s instructions. Genomic DNA was quantified using a Qubit^™^ 3.0 Fluorometer (Thermo Fisher Scientific Inc., USA). DNA libraries were prepared using the Nextera XT Library Preparation kit (Illumina Inc., USA) and sequenced on the Illumina NextSeq^™^ 500 platform (Illumina Inc., USA) using 150 bp paired-end chemistry. The raw sequence reads have been deposited in the sequence read archive under BioProject ID PRJNA1062579.

### Bioinformatics analysis

Raw sequence reads were trimmed using Trimmomatic version 0.39 and assembled *de novo* using SPAdes version 3.15.4 [19]. The sequence type (ST) of each *E. faecium* and *E. lactis* isolate was determined *in silico* using the *E. faecium* multilocus sequence typing (MLST) scheme described by Homan *et al.* [20]. Species identification was performed using the Species ID tool on the PubMLST website. Species ID uses an *in silico* ribosomal MLST approach based on 53 loci to identify the genus and species of bacterial isolates [21].

### Phylogenetic reconstruction

Single nucleotide polymorphisms (SNPs) were identified by aligning the sequence reads of each isolate to the chromosome of the *E. faecium* strain SRR24 (accession no. CP038996.1) or *E. lactis* KCTC 21015 (accession no. CP065211.1) using Snippy version 4.6.0 [22]. Phylogenetic trees were constructed using the neighbour-joining algorithm based on the SNP alignment using 200 bootstrap replicates, and the dendrogram was visualized and annotated on the interactive Tree of Life (iTOL) website [23].

### Sample preparation for spectral database construction

For the construction of the spectral database library, proteins were extracted from bacterial colonies using the ethanol/formic acid/acetonitrile protocol as recommended by the manufacturer. Briefly, pure bacterial colonies were transferred to 300 µl of HPLC-grade water using a 1 µl inoculation loop and mixed with 900 µl absolute ethanol. The suspension was then centrifuged for 2 min at 13,300 r.p.m. and the supernatant was removed. After air-drying for 5 min at room temperature, the pellet was resuspended in 25 µl of 70% formic acid and 25 µl of 100% acetonitrile, followed by centrifugation for 2 min at 13,300 r.p.m. A 1 µl volume of the supernatant was then applied to a single empty spot on the MALDI target plate and allowed to dry at room temperature. The spots were overlaid with 1 µl of α-cyano-4-hydroxycinnamic acid (HCCA) matrix solution and allowed to dry at room temperature. The target plate was loaded onto the Bruker Microflex^®^ LT/SH bench-top mass spectrometer.

### MALDI-TOF MS parameters

Analysis was performed using the Bruker Microflex^®^ LT/SH bench-top mass spectrometer (Bruker Daltonics GmbH, Bremen, Germany) with FlexControl software version 3.4. Calibration and quality control were performed using the protein extract from *E. coli* strain DH5α as a test standard. Data were obtained in automatic mode by collecting 40 laser shots with 40% laser intensity. Spectra were recorded in a positive linear mode (ion source one voltage=20.00 kV; ion source two voltage=18.20 kV; lens voltage=6.00 kV; laser frequency=60 Hz; mass range=2,000–20,000 Da).

### Analysis of raw spectra for *E. lactis* main spectrum profiles

The main spectrum profiles (MSPs) of 29 *E. lactis* isolates (the KCTC 21015 reference isolate and 28 isolates from AESOP 2021) were used to construct the in-house database. In total, 36 replicates of the spectra of each isolate were generated. Raw spectra quality was evaluated using FlexAnalysis software version 3.4 as per the manufacturer’s recommendation (Bruker Daltonics GmbH, Bremen, Germany). Baseline subtraction and smoothing were performed. Background noises, such as flatline spectra, outliers, dramatic mass shifts and anomalies, were deleted. At least 20 high-quality spectra per isolate were selected and transferred to the MBT Compass Explorer^®^ version 4.1.100 to create a single main spectrum for each *E. lactis* isolate.

### Identifying species using the in-house database

The extended direct transfer protocol commonly used by diagnostic laboratories was used for species identification. Briefly, one colony was lifted using a 1 µl sterile plastic loop and smeared as a thin film on a single empty spot on the target plate as described by Patel (2013) [24]. The spot was treated with 1 µl of 70% formic acid, allowed to dry at room temperature and overlaid with 1 µl of HCCA solution. Following a short drying period, the plate was loaded onto a Bruker Microflex^®^ LT/SH bench-top mass spectrometer. Prior to using the constructed in-house database, all isolates were identified using the BioTyper^®^ MBT Compass reference library (2022), which contains 11,897 main spectra of 4,274 species from 704 micro-organism genera.

The MALDI-TOF MS analysis results, expressed with a log score value (0.000–3.000), were indicative of the closest species matched: a score ≥2.300 was considered a high probable species identification; 2.000–2.299, a probable species identification; 1.700–1.999, a probable genus identification; and <1.700, no reliable identification. The log score value was calculated after determining the matching mass signal between the reference spectra and the unknown spectra (no match=0, complete match=1), and vice versa, and the symmetry of the matching signal intensity between the unknown and reference spectra (high symmetry yields a value close to 1, no symmetry yields a value close to 0). These three scores were then multiplied, normalized to a value of 1,000 and converted to common logarithm yielding a maximum value of 3 (i.e. log 1,000) as previously described [25].

An MSP dendrogram was generated using the MBT Compass Explorer^®^ software module version 4.1.100 and constructed using a correlation distance and average-linkage algorithm with a threshold value of 400 for a single organism. In the MSP dendrogram, the closeness of organisms is reflected by an arbitrary distance level. The distance level in an MSP dendrogram is normalized to a maximum value of 1,000.

### Statistical analysis

Using WGS as the gold standard, the sensitivity, specificity, positive predictive value (PPV) and negative predictive value (NPV) of the MALDI-TOF MS identification were calculated accordingly [26], using the following definitions: true positive – both the MALDI-TOF MS and WGS results were *E. faecium*; false positive – only the MALDI-TOF MS result was *E. faecium*; false negative – only the WGS result was *E. faecium*; and true negative – no *E. faecium* was identified by either method.

## Results

### Genomic diversity of enterococcal isolates

**Fig. 1.**
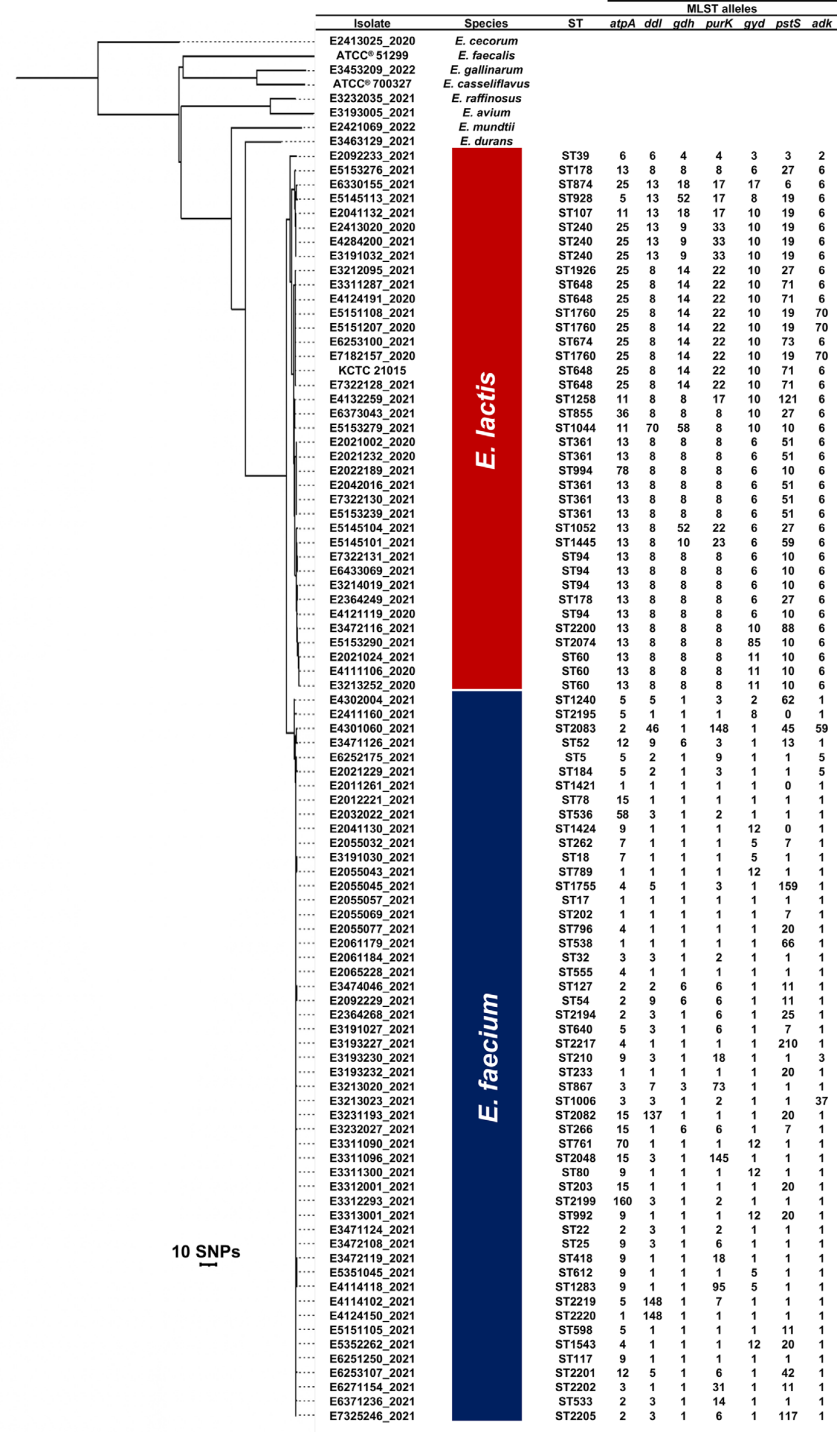
SNP-based phylogenetic tree of enterococcal genomes. The phylogeny was inferred based on 587 SNPs identified after aligning the genomes of *E. faecium* (*n*=51) (blue cluster), *E. lactis* (*n*=38) (red cluster) and non-*E. faecium* non-*E. lactis* enterococci (*n*=8) to the chromosome of the reference *E. faecium* strain SRR24 (accession no. CP038996.1) using a neighbour-joining algorithm. The non-*faecium* non-*lactis* enterococcal species included *E. avium*, *E. casseliflavus*, *E. cecorum*, *E. durans*, *E. faecalis*, *E. gallinarum*, *E. mundtii* and *E. raffinosus*. The ST and the MLST allele numbers were obtained from the PubMLST website using the MLST scheme designed by Homan *et al*. [20]. The scale bar represents the number of SNPs.

A total of 97 isolates, including 38 *E. lactis*, 51 *E. faecium* and 8 non-*E. faecium* non-*E. lactis* (NFL) enterococci, were included in the study (Table 1). Each *E. faecium* isolate (*n*=51), selected from the AESOP 2021 survey, had a unique ST. The *E. lactis* isolates included the KCTC 21015 strain and all *E. lactis* isolates misidentified as *E. faecium* in the AESOP 2020 (*n*=9) and AESOP 2021 (*n*=28) surveys. The eight NFL enterococcal isolates included two strains from the ATCC^®^ collection, *E. faecalis* ATCC^®^ 51299^™^ and *E. casseliflavus* ATCC^®^ 700327^™^, and single isolates of *E. avium*, *E. cecorum*, *E. durans*, *E. gallinarum*, *E. mundtii* and *E. raffinosus* from the AESOP surveys. The species identity of each isolate was confirmed by WGS and the SpeciesID tool. The genomes of all isolates were aligned to the chromosome of the *E. faecium* strain SRR24 and a total of 587 SNPs were identified. An SNP phylogenetic tree was constructed to visualize the relatedness of the isolates, and although closely related, all *E. lactis* isolates clustered separately from the *E. faecium* isolates (Fig. 1). A similar phylogeny was observed when the genomes were aligned to the chromosome of the *E. lactis* strain KCTC 21015 (Fig. S1, available in the online Supplementary Material).

**Table 1.** Species identification of *E. lactis*, *E. faecium* and NFL enterococci using the commercial BioTyper MBT Compass reference library (2022) before and after integrating the custom in-house database (Elac_AGAR_MF)

Sample	Source	Year	ST*	The BioTyper reference library (2022)		Elac_AGAR_MF (This study)	
				eDT†	Score	eDT†	Score
***E.lactis***							
KCTC 21015	KCTC	–	ST648	*E. faecium*	2.30	*E. lactis*	2.45
E2021002-2020	AESOP	2020	ST361	*E. faecium*	2.11	*E. lactis*	2.26
E2021232-2020	AESOP	2020	ST361	*E. faecium*	2.19	*E. lactis*	2.49
E2413020-2020	AESOP	2020	ST240	*E. faecium*	2.34	*E. lactis*	2.48
E3213252-2020	AESOP	2020	ST60	*E. faecium*	2.28	*E. lactis*	2.56
E4111106-2020	AESOP	2020	ST60	*E. faecium*	2.2	*E. lactis*	2.41
E4121119-2020	AESOP	2020	ST94	*E. faecium*	2.02	*E. lactis*	2.51
E4124191-2020	AESOP	2020	ST648	*E. faecium*	2.29	*E. lactis*	2.63
E5151207-2020	AESOP	2020	ST1760	*E. faecium*	2.28	*E. lactis*	2.51
E7182157-2020	AESOP	2020	ST1760	*E. faecium*	2.26	*E. lactis*	2.55
E2021024-2021	AESOP	2021	ST60	*E. faecium*	2.27	*E. lactis*	2.51
E2022189-2021	AESOP	2021	ST994	*E. faecium*	2.25	*E. lactis*	2.44
E2041132-2021	AESOP	2021	ST107	*E. faecium*	2.1	*E. lactis*	2.1
E2042016-2021	AESOP	2021	ST361	*E. faecium*	2.33	*E. lactis*	2.5
E2092233-2021	AESOP	2021	ST39	*E. faecium*	2.24	*E. lactis*	2.33
E2364249-2021	AESOP	2021	ST178	*E. faecium*	2.25	*E. lactis*	2.53
E3191032-2021	AESOP	2021	ST240	*E. faecium*	2.24	*E. lactis*	2.45
E3212095-2021	AESOP	2021	ST1926	*E. faecium*	2.16	*E. lactis*	2.43
E3214019-2021	AESOP	2021	ST94	*E. faecium*	2.35	*E. lactis*	2.55
E3311287-2021	AESOP	2021	ST648	*E. faecium*	2.16	*E. lactis*	2.42
E3472116-2021	AESOP	2021	ST2200	*E. faecium*	2.22	*E. lactis*	2.44
E4132259-2021	AESOP	2021	ST1258	*E. faecium*	2.36	*E. lactis*	2.53
E4284200-2021	AESOP	2021	ST240	*E. faecium*	2.27	*E. lactis*	2.47
E5145101-2021	AESOP	2021	ST1445	*E. faecium*	2.14	*E. lactis*	2.22
E5145104-2021	AESOP	2021	ST1052	*E. faecium*	2.3	*E. lactis*	2.46
E5145113-2021	AESOP	2021	ST928	*E. faecium*	2.21	*E. lactis*	2.34
E5151108-2021	AESOP	2021	ST1760	*E. faecium*	2.28	*E. lactis*	2.45
E5153239-2021	AESOP	2021	ST361	*E. faecium*	2.38	*E. lactis*	2.56
E5153276-2021	AESOP	2021	ST178	*E. faecium*	2.14	*E. lactis*	2.00
E5153279-2021	AESOP	2021	ST1044	*E. faecium*	2.12	*E. lactis*	2.53
E5153290-2021	AESOP	2021	ST2074	*E. faecium*	2.28	*E. lactis*	2.37
E6253100-2021	AESOP	2021	ST674	*E. faecium*	2.22	*E. lactis*	2.63
E6330155-2021	AESOP	2021	ST874	*E. faecium*	2.24	*E. lactis*	2.54
E6373043-2021	AESOP	2021	ST855	*E. faecium*	2.25	*E. lactis*	2.4
E6433069-2021	AESOP	2021	ST94	*E. faecium*	2.02	*E. lactis*	2.2
E7322128-2021	AESOP	2021	ST648	*E. faecium*	2.17	*E. lactis*	2.33
E7322130-2021	AESOP	2021	ST361	*E. faecium*	2.26	*E. lactis*	2.2
E7322131-2021	AESOP	2021	ST94	*E. faecium*	2.09	*E. lactis*	2.46
***E.faecium***							
E2011261-2021	AESOP	2021	ST1421	*E. faecium*	2.26	*E. faecium*	2.20
E2012221-2021	AESOP	2021	ST78	*E. faecium*	2.29	*E. faecium*	2.29
E2021229-2021	AESOP	2021	ST184	*E. faecium*	2.41	*E. faecium*	2.48
E2032022-2021	AESOP	2021	ST536	*E. faecium*	2.46	*E. faecium*	2.51
E2041130-2021	AESOP	2021	ST1424	*E. faecium*	2.45	*E. faecium*	2.44
E2055032-2021	AESOP	2021	ST262	*E. faecium*	2.5	*E. faecium*	2.52
E2055043-2021	AESOP	2021	ST789	*E. faecium*	2.47	*E. faecium*	2.50
E2055045-2021	AESOP	2021	ST1755	*E. faecium*	2.43	*E. faecium*	2.43
E2055057-2021	AESOP	2021	ST17	*E. faecium*	2.54	*E. faecium*	2.56
E2055069-2021	AESOP	2021	ST202	*E. faecium*	2.33	*E. faecium*	2.46
E2055077-2021	AESOP	2021	ST796	*E. faecium*	2.21	*E. faecium*	2.22
E2061179-2021	AESOP	2021	ST538	*E. faecium*	2.29	*E. faecium*	2.29
E2061184-2021	AESOP	2021	ST32	*E. faecium*	2.53	*E. faecium*	2.51
E2065228-2021	AESOP	2021	ST555	*E. faecium*	2.12	*E. faecium*	2.07
E2092229-2021	AESOP	2021	ST54	*E. faecium*	2.36	*E. faecium*	2.44
E2364268-2021	AESOP	2021	ST2194	*E. faecium*	2.38	*E. faecium*	2.35
E2411160-2021	AESOP	2021	ST2195	*E. faecium*	2.44	*E. faecium*	2.48
E3191027-2021	AESOP	2021	ST640	*E. faecium*	2.49	*E. faecium*	2.50
E3191030-2021	AESOP	2021	ST18	*E. faecium*	2.42	*E. faecium*	2.36
E3193227-2021	AESOP	2021	ST2217	*E. faecium*	2.51	*E. faecium*	2.48
E3193230-2021	AESOP	2021	ST210	*E. faecium*	2.47	*E. faecium*	2.51
E3193232-2021	AESOP	2021	ST233	*E. faecium*	2.38	*E. faecium*	2.34
E3213020-2021	AESOP	2021	ST867	*E. faecium*	2.42	*E. faecium*	2.42
E3213023-2021	AESOP	2021	ST1006	*E. faecium*	2.35	*E. faecium*	2.33
E3231193-2021	AESOP	2021	ST2082	*E. faecium*	2.31	*E. faecium*	2.28
E3232027-2021	AESOP	2021	ST266	*E. faecium*	2.51	*E. faecium*	2.55
E3311090-2021	AESOP	2021	ST761	*E. faecium*	2.31	*E. faecium*	2.44
E3311096-2021	AESOP	2021	ST2048	*E. faecium*	2.52	*E. faecium*	2.51
E3311300-2021	AESOP	2021	ST80	*E. faecium*	2.31	*E. faecium*	2.27
E3312001-2021	AESOP	2021	ST203	*E. faecium*	2.33	*E. faecium*	2.34
E3312293-2021	AESOP	2021	ST2199	*E. faecium*	2.52	*E. faecium*	2.49
E3313001-2021	AESOP	2021	ST992	*E. faecium*	2.41	*E. faecium*	2.45
E3471124-2021	AESOP	2021	ST22	*E. faecium*	2.27	*E. faecium*	2.44
E3471126-2021	AESOP	2021	ST52	*E. faecium*	2.23	*E. faecium*	2.32
E3472108-2021	AESOP	2021	ST25	*E. faecium*	2.53	*E. faecium*	2.56
E3472119-2021	AESOP	2021	ST418	*E. faecium*	2.53	*E. faecium*	2.54
E3474046-2021	AESOP	2021	ST127	*E. faecium*	2.45	*E. faecium*	2.43
E4114102-2021	AESOP	2021	ST2219	*E. faecium*	2.57	*E. faecium*	2.58
E4114118-2021	AESOP	2021	ST1283	*E. faecium*	2.41	*E. faecium*	2.48
E4124150-2021	AESOP	2021	ST2220	*E. faecium*	2.57	*E. faecium*	2.62
E4301060-2021	AESOP	2021	ST2083	*E. faecium*	1.73	*E. faecium*	2.03
E4302004-2021	AESOP	2021	ST1240	*E. faecium*	2.27	*E. faecium*	2.37
E5151105-2021	AESOP	2021	ST598	*E. faecium*	2.43	*E. faecium*	2.38
E5351045-2021	AESOP	2021	ST612	*E. faecium*	2.5	*E. faecium*	2.53
E5352262-2021	AESOP	2021	ST1543	*E. faecium*	2.42	*E. faecium*	2.33
E6251250-2021	AESOP	2021	ST117	*E. faecium*	2.4	*E. faecium*	2.37
E6252175-2021	AESOP	2021	ST5	*E. faecium*	2.37	*E. faecium*	2.34
E6253107-2021	AESOP	2021	ST2201	*E. faecium*	2.45	*E. faecium*	2.46
E6271154-2021	AESOP	2021	ST2202	*E. faecium*	2.57	*E. faecium*	2.56
E6371236-2021	AESOP	2021	ST533	*E. faecium*	2.41	*E. faecium*	2.43
E7325246-2021	AESOP	2021	ST2205	*E. faecium*	2.43	*E. faecium*	2.46
**Non-** ***E. faecium*** **non-** ***E. lactis*** **enterococci**							
E3193005-2021	AESOP	2021	*E. avium*	*E. avium*	2.25	*E. avium*	2.17
E2413025-2020	AESOP	2020	*E. cecorum*	*E. cecorum*	2.21	*E. cecorum*	2.15
E3463129-2021	AESOP	2021	*E. durans*	*E. durans*	2.2	*E. durans*	2.21
E3453209-2022	AESOP	2022	*E. gallinarum*	*E. gallinarum*	2.05	*E. gallinarum*	2.24
E2421069-2022	AESOP	2022	*E. mundtii*	*E. mundtii*	2.25	*E. mundtii*	2.18
E3232035-2021	AESOP	2021	*E. raffinosus*	*E. raffinosus*	2.15	*E. raffinosus*	2.22
ATCC 700327	ATCC	–	*E. casseliflavus*	*E. casseliflavus*	2.13	*E. casseliflavus*	2.15
ATCC 51299	ATCC	–	*E. faecalis*	*E. faecalis*	2.15	*E. faecalis*	2.12

*ST, sequence type;eDT, extended direct transfer..

†eDT, extended direct transfer.

Among the 38 *E. lactis* isolates, 21 STs were identified, 7 of which were represented by more than one isolate: ST361 (*n*=5), ST94 (*n*=4), ST60 (*n*=3), ST240 (*n*=3), ST648 (*n*=4), ST1760 (*n*=3) and ST178 (*n*=2). Except for *atpA* allele 5 and *gyd* allele 8, the MLST alleles harboured by *E. lactis* and *E. faecium* in this study were species-restricted. The *atpA* allele 5 was identified in seven *E. faecium* isolates (ST5, ST184, ST598, ST640, ST1240, ST2195 and ST2219) and one *E. lactis* isolate (ST928), while the *gyd* allele 8 was identified in one *E. faecium* isolate (ST2195) and one *E. lactis* isolate (ST928).

### *E. lactis* isolates are misclassified as *E. faecium* using the current Bruker commercial database

The 51 *E. faecium* and 8 NFL enterococci were correctly identified at the species level using the BioTyper^®^ MBT Compass reference library (2022). However, all *E. lactis* isolates (*n*=38) were misidentified as *E. faecium* prior to the construction of the custom in-house database (Table 1).

### Construction and validation of the Elac_AGAR_MF in-house spectral database

The custom in-house spectral database, named Elac_AGAR_MF (.btmsp file available in Supplementary Material), was constructed using the KCTC 21015 *E. lactis* isolate and the 28 *E. lactis* isolates, confirmed by WGS, from the AESOP 2021 survey [16]. To evaluate the performance and validate the constructed in-house spectral database, species identification of all 97 enterococcal isolates confirmed by WGS was re-assessed by MALDI-TOF after integrating the Elac_AGAR_MF database with the BioTyper^®^ MBT Compass reference library (2022).

**Fig. 2.**
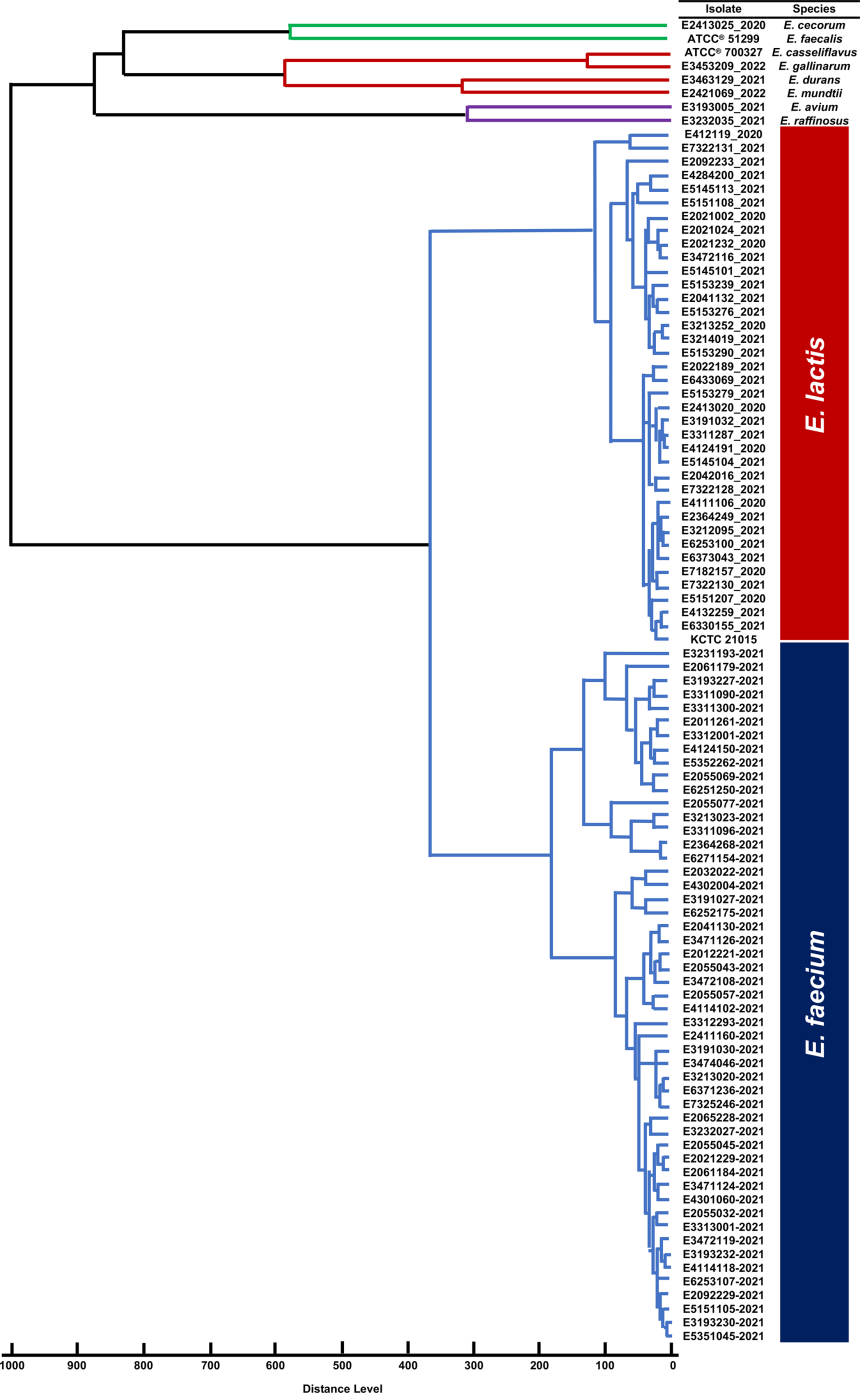
Phyloproteomic tree of enterococcal isolates based on the MSP identification of Elac_AGAR_MF using MBT Compass Explorer^®^. A total of 97 enterococcal isolates that separated into *E. faecium* cluster (*n*=51, blue), *E. lactis* cluster (*n*=38, red) and non-*E. faecium* non-*E. lactis* enterococci (*n*=8). The MSP was constructed using correlation distance, average linkage and a threshold of 400 for a single organism.

All *E. lactis* isolates (*n*=38) were accurately identified as *E. lactis* using the custom in-house database, including 84.2% (*n*=32/38) identified at the high probable species level (score ≥2.300) and 15.8% (*n*=6/38) identified at the probable species level (score 2.000–2.299). All *E. faecium* isolates (*n*=51) were accurately identified as *E. faecium*, including 84.3% (*n*=43/51) identified at the high probable species level and 15.7% (*n*=8/51) identified at the probable species level. The NFL isolates (*n*=8/8) were also accurately identified at the species level. The species identity and log score value obtained for each isolate before and after integration of the custom Elac_AGAR_MF spectral database are provided in Table 1. The phyloproteomic tree constructed based on the MSP identification using the MBT Compass Explorer^®^ software showed *E. faecium* and *E. lactis* formed two distinct clusters, separate from the cluster containing the NFL isolates (Fig. 2).

### Performance comparison before and after integrating the Elac_AGAR_MF in-house spectral database

The sensitivity, specificity, PPV and NPV for *E. faecium* identification by MALDI TOF MS using the BioTyper^®^ MBT Compass reference library (2022) were 100% (*n*=51/51), 17.4% (*n*=8/46), 57.3% (*n*=51/89) and 100% (*n*=8/8), respectively. Following the integration of the custom Elac_AGAR_MF in-house spectral database, the sensitivity, specificity, PPV and NPV for *E. faecium* identification were 100% each. Identification of *E. lactis* by MALDI-TOF MS could only be performed following the integration of the Elac_AGAR_MF custom database, and the sensitivity, specificity, PPV and NPV were 100% each (Table S1).

## Discussion

The re-classification of E*. faecium* clade B as *E. lactis* has prompted the need for diagnostic techniques, other than WGS, to accurately distinguish *E. lactis* from *E. faecium*. In this study, we have successfully constructed a database for the rapid identification of *E. lactis* using the MALDI Biotyper^®^ (Bruker Daltonics, Germany), which can be implemented in diagnostic and research laboratories. Although the re-classification of *E. faecium* clade B as a different species (i.e. *E. lactis*) has been debated, phylogenetic and phyloproteomic data from our study support the idea that *E. faecium* and *E. lactis* isolates are dissimilar enough to be characterized as two separate groups. Furthermore, our data show that the Homan *et al*. [20] *E. faecium* MLST scheme can differentiate between *E. faecium* and *E. lactis,* given that the STs of our isolates was species-specific.

Unlike the study performed by Kim *et al.* [17] in which the investigators used three *E. lactis* reference strains to construct their in-house database on the MALDI Biotyper^®^, we constructed the Elac_AGAR_MF database using 29 genetically diverse *E. lactis* isolates representing 21 different sequence types. Kim *et al.* assessed the ability of the MALDI Biotyper^®^ to differentiate enterococcal strains isolated from fermented foods, while our study included clinical strains of enterococci. Our study included clinical * E. lactis* isolates retrieved from recent bacteraemia episodes, highlighting the need not to underestimate this species’ ability to cause severe disease. The antimicrobial susceptibility profile of *E. lactis* is different to that of *E. faecium*. Unlike most *E. faecium*, *E. lactis* isolates are typically susceptible to ampicillin as they do not harbour a *pbp5*-R allele [27]. Furthermore, vancomycin resistance in *E. lactis* is rare [28]. All *E. lactis* isolates in our collection were ampicillin-susceptible and vancomycin-susceptible [16,29]. Therefore, resolving the misidentification of *E. lactis* as *E. faecium* is important to direct proper therapeutic options for *E. lactis* infections, which would include prescribing ampicillin, the treatment of choice for enterococcal infections caused by enterococci that lack mechanisms for high-level ampicillin resistance, thus limiting the overuse of vancomycin.

Two commercial MALDI-TOF MS platforms commonly used in Australian diagnostic laboratories are the MALDI Biotyper^®^ (Bruker Daltonics) and the VITEK^®^ MS (bioMérieux). Although mass spectrometry has revolutionized the field of diagnostic clinical microbiology for being a fast, reliable and inexpensive technique, the analytical performance of a mass spectrometer is significantly reliant on the diversity of reference spectra in its database. Currently, the commercial platforms do not contain an *E. lactis* reference spectrum in their database and thus misidentify *E. lactis* isolates as *E. faecium* (i.e. an ESKAPE organism), which, in the clinical setting, may lead to an unnecessary escalation in management of patients. In our study, the integration of the custom Elac_MF_AGAR database enhanced the performance of the MALDI BioTyper^®^, allowing for the accurate identification of *E. lactis* and *E. faecium* with a sensitivity and specificity value of 100%. Since we have shown the MALDI Biotyper^®^ can accurately identify *E. lactis*, a similar study is warranted to assess the ability of the VITEK^®^ MS to identify *E. lactis* to better equip our diagnostic laboratories.

While the ethanol/formic acid/acetonitrile protocol was used to prepare our custom library, the extended direct transfer protocol was used to validate the performance of the in-house library. The ethanol/formic acid/acetonitrile protocol, also known as the in-tube extraction method, yields high-quality spectra and is thus recommended for constructing custom libraries [30]. However, the extended direct transfer protocol is faster and is typically used in diagnostic laboratories to test bacterial isolates. Thus, we ensured the MALDI Biotyper^®^ yielded accurate results by validating our constructed database using the extended direct transfer protocol, allowing diagnostic laboratories to implement the Elac_AGAR_MF database onto their mass spectrometer without altering their sample preparation protocol.

Although our study accurately assigned the species of all isolates with a score >2.000, 84.3% of *E. faecium* and 84.2% of *E. lactis* were identified with a score ≥2.300 (high probable species level). The lower identification scores (score 2.000–2.299) observed in 22.7% (*n*=22/97) of isolates, while still sufficient for accurate identification, can be attributed to several factors. Variations in the amount of colony material and the homogeneity of the smear in this study can affect the spectral quality [31,32]. Factors influencing the intensities of signal peaks, such as the age of bacterial culture, concentration and location of protein in the bacterial cell as well as level of protein expression, also play a significant role in the identification score [25]. Additionally, regular calibration of the MALDI-TOF MS device is essential to maintain high spectral quality [31].

Since our study only included *E. lactis* isolated from bacteraemia cases in Australia, clinical and non-clinical *E. lactis* from other geographical locations should be tested in the future to further validate the Elac_AGAR_MF database. Furthermore, more non-*E. faecium* non-*E. lactis* enterococcal strains, as well as non-enterococcal bacterial isolates, should be tested to ensure high sensitivity and high specificity of the Elac_AGAR_MF database.

## Conclusions

We have shown that MALDI-TOF can be used to differentiate *E. lactis* from *E. faecium* in the clinical setting if *E. lactis* representatives are added to the spectral database. We urge commercial platforms to update their standard spectral database to incorporate * E. lactis* spectra to resolve the misidentification of *E. lactis* as *E. faecium* in the clinical setting where antibiotic prescription depends significantly on the accurate speciation of bacterial pathogens.

## Availability of data and materials

The datasets supporting the conclusions of this article are included within the article and the Supplementary Material. The raw sequence reads have been deposited in the sequence read archive under BioProject ID PRJNA1062579.

## supplementary material

10.1099/jmm.0.001995 Uncited Supplementary Material 1.

## Abbreviations

AESOP: Australian Enterococcal Surveillance Outcome Program
AGAR: Australian Group on Antimicrobial Resistance
ATCC: American Type Culture Collection
eDT: extended direct transfer
iTOL: interactive tree of life
KCTC: Korean Collection for Type Culture
MALDI-TOF MS: matrix-assisted laser desorption ionisation-time of flight mass spectrometry
MLST: multilocus sequence typing
MSP: main spectrum profile
NFL: non-*Enterococcus* faecium non-*Enterococcus* lactis
NPV: negative predictive value
PPV: positive predictive value
SNP: single nucleotide polymorphism
ST: sequence type
WGS: whole-genome sequencing
