# Supplementary material for: Accurate identification of Enterococcus lactis causing bacteraemia by matrix-assisted laser desorption ionization-time of flight mass spectrometry
Source: J Med Microbiol. 2025 Apr 4;74(4):001995. doi: 10.1099/jmm.0.001995 (PMC11971485; doi:10.1099/jmm.0.001995)
Supplement: Uncited Supplementary Material 1. [file jmm-74-01995-s001.pdf]

Supplementary Material

Link to Elac_AGAR_MF.btmsp database:

https://www.dropbox.com/scl/fi/tvauqqlad3np8d3utoq5g/Elac_AGAR_MF.btmsp?rlkey=8x2n64429yu0ofpa1ju4p9u7p&st=zbpagzhk&dl=0

Supplementary Table 1.

Species identified	Database	TP	FP	FN	TN	Sensitivity	Specificity
E. faecium	BioTyper® MBT Compass reference library (2022)	51	38	0	8	100%	17.4%
	BioTyper® MBT Compass reference library (2022) + Elac_AGAR_MF database	51	0	0	46	100%	100%
E. lactis	BioTyper® MBT Compass reference library (2022)	0	0	38	59	0%	100%
	BioTyper® MBT Compass reference library (2022) + Elac_AGAR_MF database	38	0	0	59	100%	100%

TP, true-positive; FP, false-positive; FN, false-negative; TN, true-negative

Supplementary Figure 1. SNP phylogeny of enterococcal isolates aligned with *E. lactis* KCTC 21015

The phylogeny was inferred based on 607 single nucleotide polymorphisms (SNPs) identified after the same set of genomes were aligned to the chromosome of the reference *E. lactis* strain KCTC 21015 (Accession number CP065211.1) using a neighbour-joining algorithm. The scale bar represents the number of SNPs. The non-*faecium* non-*lactis* enterococcal species included *E. avium*, *E. casseliflavus*, *E. cecorum*, *E. durans*, *E. faecalis*, *E. gallinarum*, *E. mundtii*, and *E. raffinosus*. The sequence type (ST) and the multilocus sequence typing (MLST) allele numbers were obtained from the PubMLST website using the MLST scheme designed by Hooman et al. (2002).

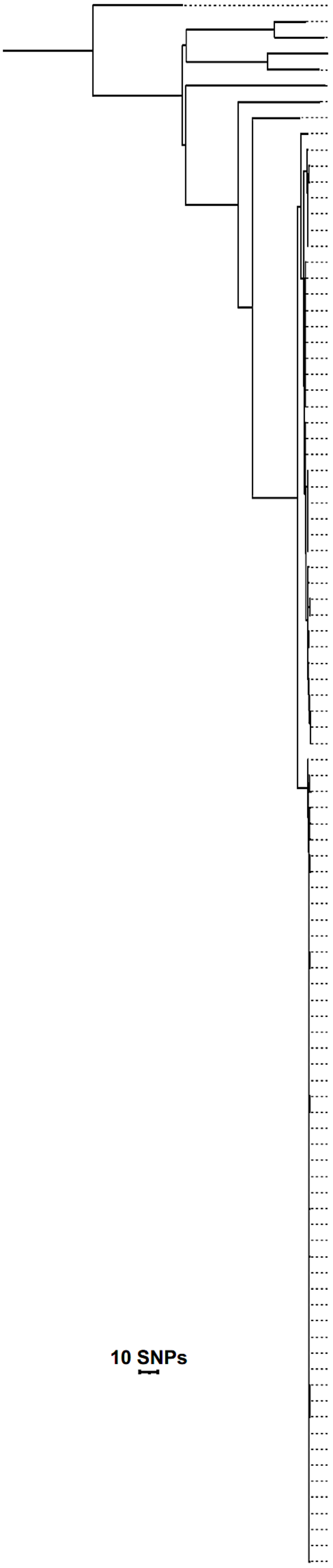

Isolate	Species	ST	MLST alleles atpA	ddl	gdh	purK	gyd	pstS	adk
E2413025_2020	*E. cecorum*								
E3232035_2021	*E. raffinosus*								
E3193005_2021	*E. avium*								
E3453209_2022	*E. gallinarum*								
ATCC® 700327	*E. casseliflavus*								
ATCC® 51299	*E. faecalis*								
E2421069_2022	*E. mundtii*								
E3463129_2021	*E. durans*								
E2092233_2021		ST39	6	6	4	4	3	3	2
E5153276_2021		ST178	13	8	8	8	6	27	6
E6330155_2021		ST874	25	13	18	17	17	6	6
E5145113_2021		ST928	5	13	52	17	8	19	6
E2041132_2021		ST107	11	13	18	17	10	19	6
E2413020_2020		ST240	25	13	9	33	10	19	6
E4284200_2021		ST240	25	13	9	33	10	19	6
E3191032_2021		ST240	25	13	9	33	10	19	6
E3212095_2021		ST1926	25	8	14	22	10	27	6
E3311287_2021		ST648	25	8	14	22	10	71	6
E4124191_2020		ST648	25	8	14	22	10	71	6
E5151108_2021		ST1760	25	8	14	22	10	19	70
E5151207_2020		ST1760	25	8	14	22	10	19	70
E6253100_2021		ST674	25	8	14	22	10	73	6
E7182157_2020		ST1760	25	8	14	22	10	19	70
E7322128_2021		ST648	25	8	14	22	10	71	6
Reference KCTC 21015		ST648	25	8	14	22	10	71	6
E4132259_2021		ST1258	11	8	8	17	10	121	6
E6373043_2021	***E. lactis***	ST855	36	8	8	8	10	27	6
E5153279_2021		ST1044	11	70	58	8	10	10	6
E2021002_2020		ST361	13	8	8	8	6	51	6
E2021232_2020		ST361	13	8	8	8	6	51	6
E2022189_2021		ST994	78	8	8	8	6	10	6
E2042016_2021		ST361	13	8	8	8	6	51	6
E7322130_2021		ST361	13	8	8	8	6	51	6
E5153239_2021		ST361	13	8	8	8	6	51	6
E5145104_2021		ST1052	13	8	52	22	6	27	6
E5145101_2021		ST1445	13	8	10	23	6	59	6
E7322131_2021		ST94	13	8	8	8	6	10	6
E6433069_2021		ST94	13	8	8	8	6	10	6
E3214019_2021		ST94	13	8	8	8	6	10	6
E2364249_2021		ST178	13	8	8	8	6	27	6
E4121119_2020		ST94	13	8	8	8	6	10	6
E3472116_2021		ST2200	13	8	8	8	10	88	6
E5153290_2021		ST2074	13	8	8	8	85	10	6
E2021024_2021		ST60	13	8	8	8	11	10	6
E4111106_2020		ST60	13	8	8	8	11	10	6
E3213252_2020		ST60	13	8	8	8	11	10	6
E2012221_2021		ST78	15	1	1	1	1	1	1
E4302004_2021		ST1240	5	5	1	3	2	62	1
E2411160_2021		ST2195	5	1	1	1	8	0	1
E3313001_2021		ST992	9	1	1	1	12	20	1
E4301060_2021		ST2083	2	46	1	148	1	45	59
E3471126_2021		ST52	12	9	6	3	1	13	1
E6252175_2021		ST5	5	2	1	9	1	1	5
E2021229_2021		ST184	5	2	1	3	1	1	5
E2011261_2021		ST1421	1	1	1	1	1	0	1
E2032022_2021		ST536	58	3	1	2	1	1	1
E2041130_2021		ST1424	9	1	1	1	12	0	1
E2055032_2021		ST262	7	1	1	1	5	7	1
E3191030_2021		ST18	7	1	1	1	5	1	1
E2055043_2021		ST789	1	1	1	1	12	1	1
E2055045_2021		ST1755	4	5	1	3	1	159	1
E2055057_2021		ST17	1	1	1	1	1	1	1
E2055069_2021		ST202	1	1	1	1	1	7	1
E2055077_2021		ST796	4	1	1	1	1	20	1
E2061179_2021		ST538	1	1	1	1	1	66	1
E2061184_2021		ST32	3	3	1	2	1	1	1
E2065228_2021		ST555	4	1	1	1	1	1	1
E3474046_2021		ST127	2	2	6	6	1	11	1
E2092229_2021		ST54	2	9	6	6	1	11	1
E2364268_2021		ST2194	2	3	1	6	1	25	1
E3191027_2021		ST640	5	3	1	6	1	7	1
E3193227_2021	***E. faecium***	ST2217	4	1	1	1	1	210	1
E3193230_2021		ST210	9	3	1	18	1	1	3
E3193232_2021		ST233	1	1	1	1	1	20	1
E3213020_2021		ST867	3	7	3	73	1	1	1
E3213023_2021		ST1006	3	3	1	2	1	1	37
E3231193_2021		ST2082	15	137	1	1	1	20	1
E3232027_2021		ST266	15	1	6	6	1	7	1
E3311090_2021		ST761	70	1	1	1	12	1	1
E3311096_2021		ST2048	15	3	1	145	1	1	1
E3311300_2021		ST80	9	1	1	1	12	1	1
E3312001_2021		ST203	15	1	1	1	1	20	1
E3312293_2021		ST2199	160	3	1	2	1	1	1
E3471124_2021		ST22	2	3	1	2	1	1	1
E3472108_2021		ST25	9	3	1	6	1	1	1
E3472119_2021		ST418	9	1	1	18	1	1	1
E5351045_2021		ST612	9	1	1	1	5	1	1
E4114118_2021		ST1283	9	1	1	95	5	1	1
E4114102_2021		ST2219	5	148	1	7	1	1	1
E4124150_2021		ST2220	1	148	1	1	1	1	1
E5151105_2021		ST598	5	1	1	1	1	11	1
E5352262_2021		ST1543	4	1	1	1	12	20	1
E6251250_2021		ST117	9	1	1	1	1	1	1
E6253107_2021		ST2201	12	5	1	6	1	42	1
E6271154_2021		ST2202	3	1	1	31	1	11	1
E7325246_2021		ST2205	2	3	1	6	1	117	1
E6371236_2021		ST533	2	3	1	14	1	1	1
